# Association of prognostic nutritional index with all-cause mortality and cardiovascular mortality: a nationwide population-based cohort study

**DOI:** 10.3389/fnut.2025.1530452

**Published:** 2025-07-14

**Authors:** Ce Zhou, You Zhou, TingYue Wang, Yun Wang, XiuYi Liang, Xin Kuang

**Affiliations:** ^1^Department of Anesthesiology, People’s Hospital of Longhua, Shenzhen, China; ^2^Shenzhen Longhua Clinical College of Medicine (People’s Hospital of Longhua), Guangdong Medical University, Shenzhen, China; ^3^The Eighth Affiliated Hospital, Sun Yat-sen University, Shenzhen, China

**Keywords:** prognostic nutritional index (PNI), mortality, NHANES, threshold effect, population-based study

## Abstract

**Background:**

The prognostic nutritional index (PNI) has shown prognostic value in various diseases, but its association with mortality in the general population remains unclear.

**Methods:**

We analyzed data from 30,741 adults in the National Health and Nutrition Examination Survey (NHANES) 2007–2018. Cox proportional hazard models examined the association between PNI and mortality outcomes. Restricted cubic spline analyses were performed to assess non-linear relationships. Subgroup analyses were conducted to evaluate effect modifications.

**Results:**

During follow-up, higher PNI values were associated with lower all-cause mortality (HR: 0.95, 95% CI: 0.94–0.96) and cardiovascular mortality (HR: 0.94, 95% CI: 0.93–0.96). Non-linear relationships were identified with threshold effects at PNI = 50.24 for all-cause mortality and PNI = 51.50 for cardiovascular mortality. The protective associations were particularly strong among participants with liver disease (*P* for interaction < 0.001).

**Conclusion:**

Prognostic nutritional index demonstrates significant predictive value for mortality outcomes in the general U.S. adult population, with identified threshold effects. These findings suggest PNI’s potential utility as a valuable risk stratification tool in clinical practice.

## 1 Introduction

Malnutrition and immune dysfunction are significant concerns in clinical practice, particularly among patients with chronic diseases and malignancies. The prognostic nutritional index (PNI), introduced by Onodera et al. (1), is crucial for assessing nutritional and immunological status using serum albumin levels and total lymphocyte counts (2, 3). PNI has demonstrated significant prognostic value across conditions like cardiovascular diseases (CVDs) and cancer (4, 5). In cardiovascular medicine, PNI is linked with adverse outcomes in heart failure patients and predicts mortality in acute cases (6). Lower PNI suggests worse outcomes, often due to malnutrition and inflammation (7, 8). Additionally, PNI predicts major adverse cardiovascular events in patients undergoing interventions (9, 10). In oncology, especially for gastrointestinal malignancies, a lower PNI correlates with poorer survival (11). It is also used to assess risk in infectious diseases, including COVID-19, as a marker for disease severity (12, 13).

Despite its widespread use, critical gaps remain in understanding the PNI and its link to mortality in the general adult population. While studies, including a recent National Health and Nutrition Examination Survey (NHANES)-based study, show lower PNI correlates with increased all-cause and cardiovascular mortality in cancer survivors (14), large-scale, population-based research is scarce. Most studies focus on specific groups, raising concerns about PNI’s applicability in the U.S. population. Research indicates lower PNI is associated with adverse outcomes in CVDs (15) and heart failure (16). Although PNI has been associated with cardiovascular outcomes in clinical settings, its relationship with all-cause and cardiovascular mortality in the general population remains, to some extent, unexplored.

The NHANES database, with its comprehensive demographic representation and long-term follow-up data, provides an ideal opportunity to address these knowledge gaps. This study aims to analyze the association of PNI with all-cause and cardiovascular mortality in a nationally representative sample of U.S. adults. Additionally, we will identify optimal PNI cut-off values for assessing mortality risk in the U.S. population while examining whether these associations vary by demographic characteristics and comorbid conditions. Our findings could provide valuable insights for public health strategies and enhance risk stratification in clinical practice.

## 2 Materials and methods

### 2.1 Study design and participants

**FIGURE 1:**
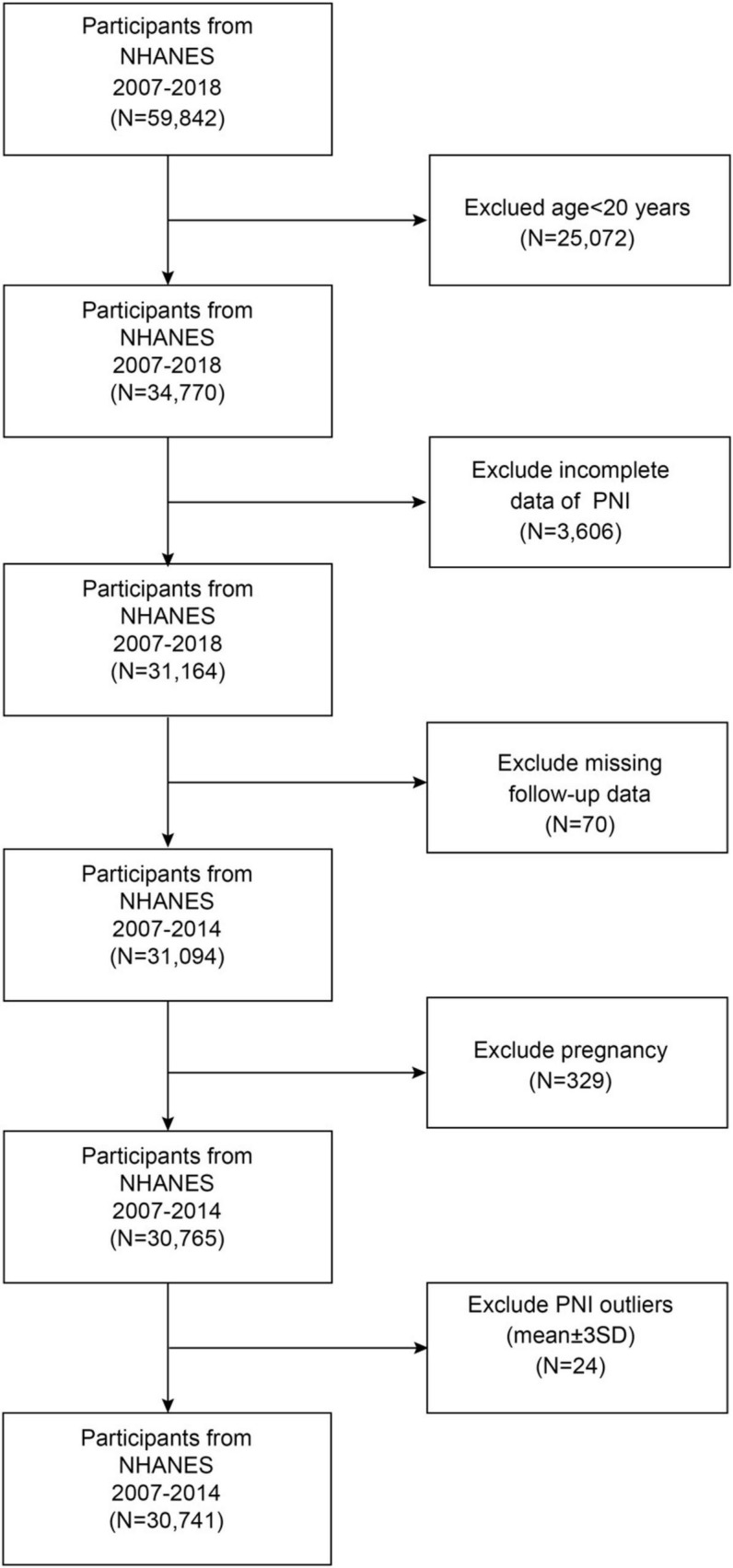
Flow chart of study participant selection.

This prospective open cohort study analyzed data from the NHANES 2007–2018, a nationally representative survey program among U.S. adults. The study design allowed participants to enter the cohort at various time points across different survey cycles (2007–2018), with baseline data collected during NHANES examinations and mortality outcomes ascertained through linkage to the National Death Index (NDI) through 31 December 2019. This temporal framework enables the assessment of exposure-outcome relationships, with PNI measured at baseline and subsequent mortality events tracked prospectively over a median follow-up period of 7.25 years. The participant selection process is illustrated in Figure 1. From an initial sample of 59,842 NHANES participants, we established our study population through a systematic screening process. We excluded participants aged < 20 years (*n* = 25,072), those with incomplete PNI data (*n* = 3,606), those with missing follow-up information (*n* = 70), pregnant individuals (*n* = 329), and PNI outliers (mean ± 3 SD; *n* = 24), resulting in a final analytical sample of 30,741 participants. All data used in this study are publicly accessible through the NHANES database^1^.

### 2.2 Assessment of PNI

The PNI (17) was calculated using the following formula:


PNI=serumalbumin(g/L)+5×lymphocytecount(10/9L).

The serum albumin levels were measured using the bromocresol violet (BCP) method. At the same time, lymphocyte counts were obtained through complete blood count (CBC) analysis using a Beckman Coulter Automated Hematology Analyzer DxH 900 (Beckman-Coulter, Brea, CA, USA). The CBC analysis was performed following standardized NHANES protocols. Higher PNI values indicate better nutritional status.

### 2.3 All-cause and cardiovascular mortality

Assessment of mortality outcomes was conducted through comprehensive data linkage with the NDI records, with follow-up extending through 31 December 2019. The follow-up duration for each study participant was calculated from their initial NHANES Mobile Examination Center (MEC) examination date until either the date of death or the end of the follow-up period (31 December 2019), whichever occurred first. All-cause mortality encompassed deaths from any cause during the follow-up period, while cardiovascular mortality was specifically identified using the International Classification of Diseases, 10th Revision (ICD-10) coding system. Cardiovascular-related deaths were defined by ICD-10 codes, including acute rheumatic fever and chronic rheumatic heart diseases (I00–I09), hypertensive heart disease (I11), hypertensive heart and renal disease (I13), ischemic heart diseases and other forms of heart disease (I20–I51), and cerebrovascular diseases (I60–I69).

### 2.4 Definition of covariates

Sociodemographic and lifestyle factors were systematically collected during the baseline examination. Demographic variables included age, gender, race/ethnicity (categorized as Mexican American people, other Hispanic people, non-Hispanic White people, non-Hispanic Black people, and others), educational attainment (less than high school, high school or equivalent, and college or higher), marital status (married/cohabiting, widowed/divorced/separated, and never married), and family income-to-poverty ratio. Lifestyle factors encompassed sedentary behavior (<3, 3–6, and >6 h/day), smoking status (current, former, and never), and alcohol consumption patterns (non-drinker, <5, 5–10, and ≥10 drinks/month). Medical conditions were assessed through self-reported diagnoses, medication use, and clinical measurements. CVD was identified through self-reported physician-confirmed diagnoses of congestive heart failure, coronary heart disease, angina pectoris, myocardial infarction, or stroke. Hypertension was defined by meeting any of the following criteria: self-reported physician diagnosis, current use of antihypertensive medications, or measured blood pressure ≥ 130/80 mmHg. Diabetes was identified through self-reported diagnosis, use of insulin or antidiabetic medications, or laboratory values meeting established diagnostic criteria (fasting plasma glucose ≥ 7.0 mmol/L, 2-h postprandial glucose ≥ 200 mg/dl, or HbA1c ≥ 6.5%). Chronic obstructive pulmonary disease (COPD) status was determined based on self-reported physician diagnosis of chronic bronchitis, emphysema, or COPD. Depressive symptoms were evaluated using the Patient Health Questionnaire-9 (PHQ-9); clinically significant depression was defined as a PHQ-9 score ≥ 10. Additional covariates included body mass index (calculated from measured height and weight), presence of liver conditions and cancer (self-reported), high cholesterol (based on diagnosis and medication use), and use of medications affecting lipid or glucose profiles. The estimated glomerular filtration rate (eGFR) was estimated using the Chronic Kidney Disease Epidemiology Collaboration (CKD-EPI) equation.

### 2.5 Statistical analyses

Statistical analyses were conducted according to the National Center for Health Statistics guidelines, considering the survey weights required for the complex sampling design used in NHANES. The data were presented as survey-weighted mean [95% confidence intervals (CIs)] for continuous variables and survey-weighted percentage (95% CIs) for categorical variables. *P* values were calculated using survey-weighted linear regression for continuous variables and survey-weighted Chi-square tests for categorical variables. Missing data were addressed through random forest imputation to minimize potential bias. The PNI was divided into quartiles for the primary analyses. Survival curves were generated using the Kaplan–Meier method to estimate cumulative event-free survival probabilities across PNI quartile groups. The between-group differences were then assessed using the log-rank test. To examine the association between PNI and mortality outcomes, three Cox proportional hazards regression models were constructed: an unadjusted model (model 1), a demographic-adjusted model controlling for age, sex, and race/ethnicity (model 2), and a fully adjusted model (model 3). Model 3 incorporated a comprehensive range of demographic factors, including age, sex, race/ethnicity, education level, marital status, and income-to-poverty ratio. Additionally, lifestyle factors such as sedentary behavior, smoking status, and alcohol consumption were considered. Clinical measurements were also included, such as body mass index (BMI), hemoglobin, alanine aminotransferase (ALT), eGFR, and urine albumin-to-creatinine ratio (UACR). Furthermore, comorbidities, including cancer, depression, hypertension, COPD, dyslipidemia, diabetes, liver disease, CVD, and hyperuricemia were taken into account. Potential non-linear relationships were evaluated using restricted cubic spline analyses. Following confirmation of the existence of non-linear associations, two-piecewise Cox proportional hazards regression models were employed to identify threshold effects, with optimal threshold points determined through likelihood ratio tests. To assess the generalizability of our findings across diverse population subgroups, we conducted stratified analyses using the fully adjusted model (model 3). The prespecified subgroups included demographic characteristics [sex, age, race/ethnicity, education level, marital status, and ratio of family income to poverty (RIP)], and lifestyle factors (BMI, smoking status, and alcohol consumption). Additionally, the following clinical parameters were considered: UACR and eGFR. The presence or absence of comorbidities was also taken into account, including hypertension, COPD, dyslipidemia, diabetes, liver disease, cancer and malignancy, CVD, hyperuricemia, and depression. Interaction tests were conducted to ascertain the extent of heterogeneity in the associations between these subgroups. All analyses were conducted using R software, version 4.3.1 (The R Foundation),^2^ and Empower (R) (X&Y Solutions, Inc., Boston, MA, USA). The threshold for statistical significance was set at a two-sided *P*-value of less than 0.05.

## 3 Results

### 3.1 Baseline characteristics of the participants

The median follow-up time was 7.25 years (interquartile range: 4.17–10.17 years) for the entire cohort of 30,741 participants. Table 1 presents the study population’s baseline characteristics, categorized by PNI quartiles. The study’s average participant age was represented across the quartiles, with non-Hispanic White people making up most of the cohort. Participants in the lowest PNI quartile (Q1) were generally older, more likely to be female, and non-Hispanic Black people than those in the highest PNI quartile (Q4). They were also associated with lower income-to-expense ratios and higher BMI. Additionally, Q1 participants exhibited a higher prevalence of chronic conditions like hypertension, diabetes, and CVD. While lower quartile participants were less likely to consume alcohol, the frequency of current smoking increased in higher quartiles, demonstrating a statistically significant variation across groups.

**TABLE 1:** Basic characteristics of study participants stratified by PNI, weighted.

Variable	PNI				*P*-value
	Q1 (20.00–49.50)	Q2 (50.00–52.50)	Q3 (53.00–55.50)	Q4 (56.00–90.00)	
Age	55.3 (54.5, 56.0)	49.6 (49.0, 50.2)	46.1 (45.6, 46.7)	41.8 (41.3, 42.4)	<0.001
RIP	3.0 (2.9, 3.0)	3.1 (3.0, 3.2)	3.1 (3.0, 3.2)	2.9 (2.8, 3.0)	<0.001
BMI (kg/m^2^)	30.3 (30.1, 30.6)	29.3 (29.1, 29.6)	28.6 (28.4, 28.9)	28.3 (28.1, 28.5)	<0.001
UACR	76.5 (64.4, 88.6)	27.5 (23.7, 31.4)	21.6 (18.8, 24.3)	17.1 (14.8, 19.3)	<0.001
Lymphocyte count (1,000 cells/μl)	1.6 (1.5, 1.6)	1.9 (1.9, 1.9)	2.2 (2.1, 2.2)	2.8 (2.8, 2.9)	<0.001
Serum albumin (g/L)	39.3 (39.2, 39.4)	41.9 (41.8, 42.0)	43.5 (43.4, 43.6)	45.2 (45.1, 45.3)	<0.001
ALT (U/L)	22.8 (22.3, 23.3)	24.1 (23.5, 24.6)	25.8 (25.3, 26.3)	27.6 (27.0, 28.1)	<0.001
eGFR (ml/min/1.73 m^2^)	87.1 (86.1, 88.1)	93.2 (92.3, 94.0)	96.1 (95.3, 96.8)	98.9 (98.1, 99.7)	<0.001
Hemoglobin (g/dl)	13.6 (13.6, 13.7)	14.1 (14.0, 14.1)	14.4 (14.3, 14.4)	14.7 (14.6, 14.7)	<0.001
PNI	47.08 (47.01, 47.16)	51.29 (51.27, 51.31)	54.22 (54.20, 54.24)	59.01 (58.92, 59.11)	<0.001
Sex, %					<0.001
Male	39.9 (38.4, 41.4)	44.6 (43.1, 46.2)	50.5 (49.1, 51.8)	57.9 (56.6, 59.2)	
Female	60.1 (58.6, 61.6)	55.4 (53.8, 56.9)	49.5 (48.2, 50.9)	42.1 (40.8, 43.4)	
Race, %					<0.001
Mexican American people	6.5 (5.3, 7.9)	8.0 (6.7, 9.6)	9.3 (7.8, 11.2)	10.2 (8.4, 12.3)	
Other Hispanic people	5.2 (4.2, 6.3)	5.9 (4.9, 7.1)	5.8 (4.9, 6.9)	6.5 (5.4, 7.7)	
Non-Hispanic White people	68.0 (65.1, 70.9)	67.4 (64.4, 70.2)	66.5 (63.4, 69.4)	65.7 (62.5, 68.8)	
Non-Hispanic Black people	14.1 (12.4, 16.0)	10.5 (9.0, 12.3)	9.9 (8.6, 11.3)	8.6 (7.4, 10.0)	
Other race	6.2 (5.4, 7.2)	8.1 (7.0, 9.3)	8.5 (7.4, 9.7)	9.0 (7.9, 10.2)	
Education level, %					0.018
Lower than high school	16.7 (15.3, 18.2)	14.7 (13.4, 16.2)	15.3 (13.9, 16.9)	16.6 (15.1, 18.3)	
High school or equivalent	22.2 (21.0, 23.6)	22.5 (20.9, 24.1)	23.3 (21.8, 24.8)	23.8 (22.2, 25.4)	
College or above	61.0 (59.0, 63.0)	62.8 (60.6, 65.0)	61.4 (59.2, 63.5)	59.6 (57.2, 62.0)	
Marital status, %					<0.001
Married/cohabiting	61.8 (60.1, 63.4)	65.1 (63.3, 66.9)	63.8 (62.0, 65.6)	62.5 (60.8, 64.2)	
Widowed/divorced/separated	24.7 (23.4, 25.9)	19.4 (18.1, 20.7)	16.8 (15.8, 17.9)	14.7 (13.7, 15.9)	
Never married	13.6 (12.5, 14.7)	15.5 (14.2, 16.9)	19.4 (17.8, 21.0)	22.7 (21.0, 24.6)	
Sedentary time, %					0.77
<3 h/day	13.2 (12.2, 14.3)	13.0 (12.1, 13.9)	13.8 (12.7, 15.0)	13.1 (12.0, 14.3)	
3–6 h/day	45.9 (44.2, 47.6)	46.3 (44.6, 48.0)	45.2 (43.5, 47.0)	46.7 (45.2, 48.3)	
>6 h/day	41.0 (39.1, 42.9)	40.7 (39.1, 42.4)	41.0 (39.1, 42.8)	40.2 (38.4, 41.9)	
Alcohol consumption, %					<0.001
Never	17.3 (16.0, 18.8)	15.2 (13.8, 16.7)	12.7 (11.6, 13.8)	12.9 (11.5, 14.3)	
0 to <5 drinks/month	42.5 (40.8, 44.2)	39.3 (37.3, 41.3)	40.0 (38.0, 42.0)	35.8 (34.1, 37.6)	
5 to < 10 drinks/month	9.0 (7.9, 10.2)	11.1 (9.9, 12.5)	10.7 (9.6, 11.9)	12.0 (10.8, 13.3)	
≥10 drinks/month	31.1 (29.6, 32.8)	34.3 (32.5, 36.2)	36.6 (34.6, 38.7)	39.3 (37.4, 41.3)	
Smoking status, %					<0.001
Never smoked	56.9 (54.9, 58.9)	57.9 (56.0, 59.7)	56.1 (54.3, 57.8)	52.2 (50.6, 53.9)	
Former smoker	28.9 (27.2, 30.8)	25.7 (24.1, 27.4)	23.7 (22.2, 25.2)	21.6 (20.4, 22.8)	
Current smoker	14.2 (13.1, 15.4)	16.4 (15.2, 17.7)	20.2 (18.8, 21.7)	26.2 (24.6, 27.8)	
Cancer and malignancy, %					<0.001
No	84.2 (83.0, 85.4)	89.0 (88.0, 89.9)	91.8 (91.0, 92.6)	93.1 (92.2, 93.8)	
Yes	15.8 (14.6, 17.0)	11.0 (10.1, 12.0)	8.2 (7.4, 9.0)	6.9 (6.2, 7.8)	
Depression, %					0.007
No	91.0 (90.0, 91.8)	92.0 (91.1, 92.9)	93.0 (92.2, 93.8)	92.0 (91.1, 92.8)	
Yes	9.0 (8.2, 10.0)	8.0 (7.1, 8.9)	7.0 (6.2, 7.8)	8.0 (7.2, 8.9)	
Hypertension, %					<0.001
No	44.3 (42.5, 46.1)	50.8 (49.1, 52.5)	55.9 (54.2, 57.5)	56.4 (54.7, 58.0)	
Yes	55.7 (53.9, 57.5)	49.2 (47.5, 50.9)	44.1 (42.5, 45.8)	43.6 (42.0, 45.3)	
COPD, %					<0.001
No	89.1 (87.7, 90.4)	92.7 (91.7, 93.6)	93.0 (92.1, 93.8)	94.2 (93.3, 95.0)	
Yes	10.9 (9.6, 12.3)	7.3 (6.4, 8.3)	7.0 (6.2, 7.9)	5.8 (5.0, 6.7)	
Hyperlipidemia, %					0.7876
No	27.5 (26.1, 28.9)	27.7 (26.1, 29.3)	27.3 (25.7, 28.9)	28.2 (26.8, 29.6)	
Yes	72.5 (71.1, 73.9)	72.3 (70.7, 73.9)	72.7 (71.1, 74.3)	71.8 (70.4, 73.2)	
Diabetes, %					<0.001
No	79.63 (78.37, 80.83)	85.61 (84.39, 86.75)	86.81 (85.65, 87.89)	88.96 (88.08, 89.78)	
Yes	20.37 (19.17, 21.63)	14.39 (13.25, 15.61)	13.19 (12.11, 14.35)	11.04 (10.22, 11.92)	
Liver disease, %					0.001
No	95.2 (94.3, 96.0)	96.4 (95.8, 96.9)	96.8 (96.2, 97.4)	96.7 (96.2, 97.1)	
Yes	4.8 (4.0, 5.7)	3.6 (3.1, 4.2)	3.2 (2.6, 3.8)	3.3 (2.9, 3.8)	
CVD, %					<0.001
No	84.4 (83.1, 85.6)	91.4 (90.6, 92.3)	93.1 (92.2, 93.9)	94.8 (94.2, 95.4)	
Yes	15.6 (14.4, 16.9)	8.6 (7.7, 9.4)	6.9 (6.1, 7.8)	5.2 (4.6, 5.8)	
Hyperuricemia, %					0.0015
No	81.3 (79.9, 82.6)	83.6 (82.5, 84.6)	83.2 (81.8, 84.5)	80.8 (79.5, 82.0)	
Yes	18.7 (17.4, 20.1)	16.4 (15.4, 17.5)	16.8 (15.5, 18.2)	19.2 (18.0, 20.5)	
All-cause mortality, %					<0.001
Alive	86.5 (85.4, 87.6)	93.5 (92.8, 94.2)	95.3 (94.7, 95.9)	96.2 (95.7, 96.6)	
Dead	13.5 (12.4, 14.6)	6.5 (5.8, 7.2)	4.7 (4.1, 5.3)	3.8 (3.4, 4.3)	
Cardiovascular mortality, %					<0.001
Alive	95.83 (95.24, 96.35)	98.21 (97.75, 98.58)	98.56 (98.23, 98.83)	99.22 (99.01, 99.38)	
Dead	4.17 (3.65, 4.76)	1.79 (1.42, 2.25)	1.44 (1.17, 1.77)	0.78 (0.62, 0.99)	

In this study, missing data were observed across several variables. Specifically, the number and proportion of missing data were as follows: 35 missing values (0.11%) for education level, 14 missing values (0.05%) for marital status, 2,876 missing values (9.35%) for income to expense ratio, 386 missing values (1.26%) for BMI, 26 missing values (0.08%) for cancer and malignancy, 2,904 missing values (9.44%) for depression, 467 missing values (1.52%) for UACR, 169 missing values (0.55%) for sedentary time, 7,554 missing values (24.56%) for alcohol consumption, 14 missing values (0.05%) for ALT, 1 missing value (0.003%) for hypertension, 3 missing values (0.01%) for COPD, 1 missing value (0.003%) for hyperlipidemia, 2 missing values (0.01%) for eGFR, 55 missing values (0.18%) for liver disease, 4 missing values (0.01%) for cardiovascular disease, 13 missing values (0.04%) for hyperuricemia, and 23 missing values (0.07%) for smoking status. BMI, body mass index; RIP, ratio of family income to poverty; CVD, cardiovascular disease; COPD, chronic obstructive pulmonary disease; UACR, urine albumin-to-creatinine ratio; ALT, alanine aminotransferase; eGFR, estimated glomerular filtration rate.

### 3.2 Association between PNI and mortality

**FIGURE 2:**
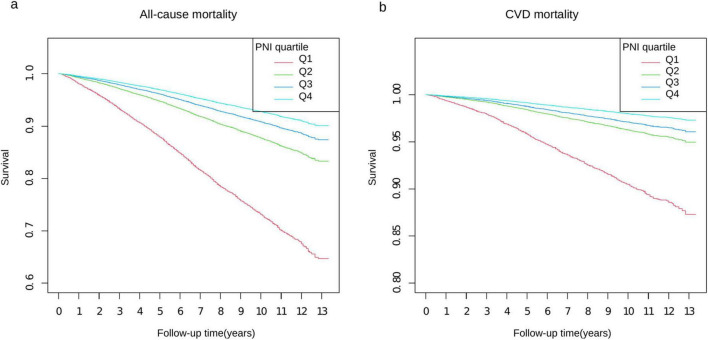
Kaplan–Meier survival curves stratified by PNI quartiles. Panel **(a)** shows all-cause mortality and panel **(b)** shows CVD mortality over 13 years of follow-up. The *y*-axis represents survival probability, with panel **(a)** ranging from 0.6 to 1.0 and panel **(b)** from 0.80 to 1.0. Four PNI quartiles (Q1–Q4) are represented by different colored lines: Q1 (red), Q2 (green), Q3 (blue), and Q4 (light blue). The *x*-axis shows the follow-up time in years from 0 to 13.

Survival curve analysis demonstrated a significant increase in survival rates with higher PNI values in both all-cause and cardiovascular mortality (*P* < 0.0001) (Figure 2). This pattern was consistently observed in our chronic kidney disease (CKD) subgroup analysis, where higher PNI quartiles showed superior survival outcomes over 13 years of follow-up (Supplementary Figure 1).

Table 2 presents the associations between PNI and mortality outcomes across three increasingly adjusted models. Our findings demonstrate robust and consistent relationships between PNI and both all-cause and cardiovascular mortality, with evidence of dose-dependent protective effects.

**TABLE 2:** Association of PNI with all-cause and CVD mortality using weighted Cox proportional hazards models, weighted.

Characteristics	Continuous	Quartiles of PNI				*P* for trend
	Per unit	Q1	Q2	Q3	Q4	
**All-cause mortality**						
Model 1	0.87 (0.86–0.88)	Reference	0.43 (0.37–0.50)	0.30 (0.25–0.35)	0.24 (0.20–0.29)	<0.001
Model 2	0.93 (0.92–0.94)	Reference	0.56 (0.47–0.66)	0.44 (0.36–0.54)	0.32 (0.26–0.39)	<0.001
Model 3	0.95 (0.94–0.96)	Reference	0.69 (0.60–0.80)	0.57 (0.47–0.69)	0.40 (0.33–0.50)	<0.001
**CVD mortality**						
Model 1	0.84 (0.83–0.86)	Reference	0.30 (0.23–0.38)	0.17 (0.15–0.20)	0.16 (0.13–0.21)	<0.001
Model 2	0.92 (0.91–0.94)	Reference	0.63 (0.49–0.79)	0.63 (0.53–0.74)	0.51 (0.41–0.63)	<0.001
Model 3	0.94 (0.93–0.96)	Reference	0.69 (0.55–0.88)	0.69 (0.55–0.86)	0.55 (0.43–0.69)	<0.001

Model 1 adjusted for: none. Model 2 adjusted for gender, age, and race. Model 3 adjusted for sex, age, race/ethnicity, education level, marital status, RIP, BMI, cancer and malignancy, depression, UACR, sedentary time, alcohol consumption, hypertension, chronic obstructive pulmonary disease, dyslipidemia, eGFR, diabetes, liver disease, CVD, hemoglobin, hyperuricemia, smoking status, and ALT.

In the analysis of all-cause mortality, the fully adjusted model (model 3) demonstrated that each unit increase in PNI was associated with a 5% reduction in mortality risk (HR: 0.95, 95% CI: 0.94–0.96, *P* < 0.001). To further characterize this relationship, we performed quartile analysis, which revealed a significant dose-response relationship (*P* for trend < 0.001): with Q1 as a reference, the HRs were 0.69 (95% CI: 0.60–0.80) for Q2, 0.57 (95% CI: 0.47–0.69) for Q3, and 0.40 (95% CI: 0.33–0.50) for Q4.

For cardiovascular mortality, the fully adjusted model showed that each unit increase in PNI was associated with a 6% reduction in mortality risk (HR: 0.94, 95% CI: 0.93–0.96, *P* < 0.001). Quartile analysis also demonstrated a significant dose-response relationship (*P* for trend < 0.001): with Q1 as a reference, the HRs were 0.69 (95% CI: 0.55–0.88) for Q2, 0.69 (95% CI: 0.55–0.86) for Q3, and 0.55 (95% CI: 0.43–0.69) for Q4.

The effect estimates attenuated from the crude model to the fully adjusted model: for all-cause mortality, the HR weakened from 0.87 (95% CI: 0.86–0.88) to 0.95 (95% CI: 0.94–0.96), and for cardiovascular mortality, from 0.84 (95% CI: 0.83–0.86) to 0.94 (95% CI: 0.93–0.96). However, all associations remained statistically significant across all models (all *P* < 0.001).

### 3.3 Dose-response relationship between PNI and mortality

**FIGURE 3:**
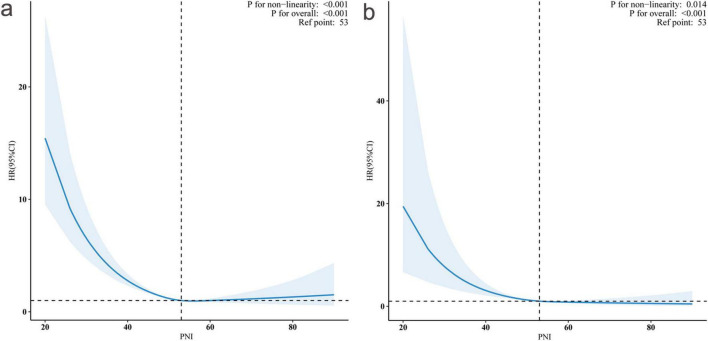
Non-linear association between prognostic nutritional index (PNI) and hazard ratios (HRs) for all-cause and cardiovascular mortality. Restricted cubic spline curves showing the relationship between PNI and adjusted HRs with 95% confidence intervals (shaded areas). Panel **(a)** demonstrates the association with all-cause mortality (*P* for non-linearity < 0.001), and panel **(b)** shows cardiovascular mortality (*P* for non-linearity = 0.014). The reference point was PNI = 53 (vertical dashed lines, HR = 1.0). Both outcomes showed significant overall associations (*P* < 0.001 for both).

As illustrated in Figure 3, restricted cubic spline analyses revealed significant non-linear associations between PNI and mortality outcomes (*P* for non-linearity < 0.001 for both all-cause and cardiovascular mortality) after adjusting for potential confounders.

We conducted threshold effect analyses using two-piecewise Cox proportional hazards regression models to explore potential non-linear relationships between PNI and mortality outcomes (Table 3). Our results revealed distinct inflection points in the association between PNI and both mortality outcomes, with significant threshold effects confirmed by log-likelihood ratio tests (both *P* < 0.001).

**TABLE 3:** Threshold effect analysis of PNI and all-cause mortality and CVD mortality.

Outcome	Adjusted HR (95% CI), *P*-value
**All-cause mortality**	
Inflection point	50.24
PNI < inflection point	0.916 (0.889, 0.943), <0.001
PNI > inflection point	0.994 (0.978, 1.011), 0.4774
Log-likelihood ratio	<0.001
**CVD Mortality**	
Inflection point	51.5
PNI < inflection point	0.912 (0.882, 0.943), <0.001
PNI > inflection point	0.963 (0.912, 1.001), 0.38
Log-likelihood ratio	<0.001

Sex, age, race/ethnicity, education level, marital status, RIP, BMI, cancer and malignancy, depression, UACR, sedentary time, alcohol consumption, hypertension, COPD, dyslipidemia, eGFR, diabetes, liver disease, CVD, hemoglobin, hyperuricemia, smoking status, and ALT were adjusted.

For all-cause mortality, we identified an inflection point at PNI = 50.24. Below this threshold, each unit increase in PNI was associated with an 8.4% reduction in mortality risk (HR: 0.916, 95% CI: 0.889–0.943, *P* < 0.001), indicating a strong protective effect. However, above this threshold, the association became statistically non-significant (HR: 0.994, 95% CI: 0.978–1.011, *P* = 0.4774). This pattern implies that the protective effect of PNI against all-cause mortality may reach its maximum benefit at approximately 50.24 units.

Similarly, the analysis identified an inflection point at PNI = 51.50 for cardiovascular mortality. In the lower PNI range (<51.50), each unit increase was associated with an 8.8% reduction in cardiovascular mortality risk (HR: 0.912, 95% CI: 0.882–0.943, *P* < 0.001). Beyond this threshold, the association weakened and became non-significant (HR: 0.963, 95% CI: 0.912–1.001, *P* = 0.38).

These findings were further validated in our supplementary analysis of CKD patients. The Supplementary material demonstrates consistent non-linear associations between PNI and mortality outcomes in this high-risk subpopulation, with similar threshold patterns observed (Supplementary Figure 2).

### 3.4 Subgroup analyses

**FIGURE 4:**
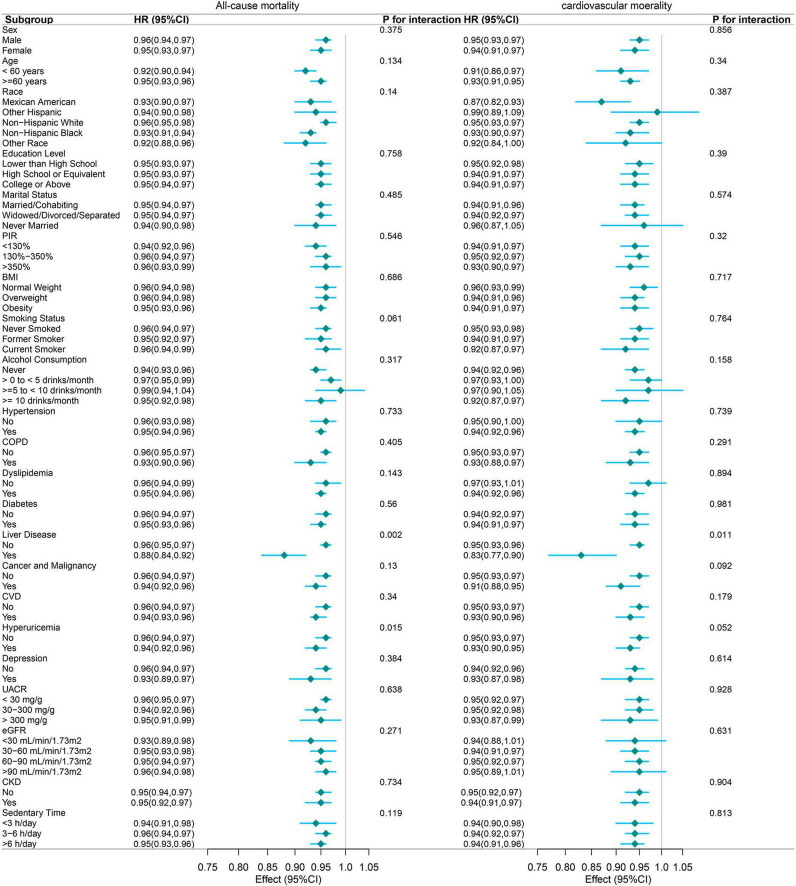
Forest plot of subgroup analyses examining the association between PNI (per 1-unit increase) and mortality outcomes. The following variables were adjusted in the study, except for the specific variable being analyzed in each subgroup: Sex, age, race/ethnicity, education level, marital status, RIP, BMI, cancer and malignancy, depression, UACR, sedentary time, alcohol consumption, hypertension, COPD, dyslipidemia, eGFR, diabetes, liver disease, cardiovascular disease, hemoglobin, hyperuricemia, smoking status, and ALT.

Figure 4 shows the subgroup analyses conducted to evaluate the consistency of the association between the PNI and mortality outcomes across various population characteristics. For all-cause mortality, the protective association of PNI was largely consistent across most subgroups. Significant effect modifications were observed for liver disease (*P* for interaction = 0.002) and hyperuricemia (*P* for interaction = 0.015). Notably, the association was more pronounced among participants with liver disease (HR: 0.88, 95% CI: 0.84–0.92) than those without liver disease (HR: 0.96, 95% CI: 0.95–0.97). Similarly, the protective association differed by hyperuricemia status, with slightly stronger effects observed in participants with hyperuricemia (HR: 0.94, 95% CI: 0.92–0.96) compared to those without (HR: 0.96, 95% CI: 0.94–0.97). This suggests that patients with hyperuricemia might benefit more from improved nutritional status, possibly due to the complex interactions between uric acid metabolism, inflammation, and nutritional status. This also suggests a potentially heightened protective effect of PNI in individuals with compromised liver function, possibly due to the nutritional and metabolic challenges associated with liver disease.

For cardiovascular mortality, the protective association was similarly consistent across subgroups, with liver disease again emerging as the sole significant effect modifier (*P* for interaction = 0.011). Participants with liver disease exhibited a stronger protective association (HR: 0.83, 95% CI: 0.77–0.90) compared to those without (HR: 0.96, 95% CI: 0.93–0.96). This finding underscores the potential importance of nutritional status in mitigating cardiovascular risk among patients with liver disease.

The robustness of these associations was evident across various demographic characteristics, including age, sex, race, education level, and marital status. Furthermore, the associations remained consistent across vital clinical parameters, such as hypertension, diabetes, CVD, COPD, and different categories of eGFR, and CKD.

While the magnitude of association varied slightly across different subgroups, the protective effect of higher PNI values remained statistically significant in most stratified analyses, with hazard ratios consistently below 1.00. This consistent pattern suggests that the protective association between PNI and mortality outcomes is largely independent of demographic and clinical characteristics, supporting its potential utility as a robust prognostic indicator across diverse patient populations. Future research should explore the underlying mechanisms driving these associations, particularly in subgroups with significant effect modifications.

## 4 Discussion

This large-scale, population-based study of 30,741 U.S. adults revealed several important findings regarding the association between PNI and mortality outcomes. First, we demonstrated a robust inverse association of PNI with all-cause and cardiovascular mortality, with each unit increase in PNI associated with a 5% and 6% reduction in risk, respectively. Second, we identified significant non-linear relationships and critical threshold points in these associations: PNI of 50.24 for all-cause mortality and 51.50 for cardiovascular mortality, beyond which the protective effects plateaued. Third, the protective association was particularly pronounced among participants with liver disease, suggesting a potentially heightened importance of nutritional status in this subgroup. Fourth, quartile analyses revealed a clear dose-response relationship, with individuals in the highest PNI quartile showing 60% lower all-cause mortality risk and 45% lower cardiovascular mortality risk compared to those in the lowest quartile. These associations remained robust after comprehensive adjustment for potential confounders and were largely consistent across various demographic and clinical subgroups, supporting PNI’s potential utility as a valuable prognostic indicator in the general U.S. adult population.

Our findings align with several recent studies examining the prognostic value of PNI while offering unique insights through our larger general population focus. Yu et al. (18) demonstrated that PNI was inversely associated with all-cause mortality in CKD patients, identifying a critical threshold of 50.5, similar to our threshold of 50.24 for all-cause mortality. This result is generally consistent with the findings based on our CKD population (Supplementary Figures 1, 2). Interestingly, in our study, the effect of PNI on all-cause mortality was consistent between CKD and non-CKD populations. Our supplementary analyses in CKD patients (Supplementary Figures 1, 2) provide robust validation of our main findings in a high-risk subpopulation particularly vulnerable to nutritional and immune dysfunction. The consistency of protective associations between CKD and non-CKD populations underscores PNI’s broad clinical applicability as a prognostic tool across diverse patient populations. The similar threshold values observed in both the general population and CKD subgroup further support the clinical utility of our identified PNI cut-off points for risk stratification. Through analyzing data from 14,349 NHANES and NDI subjects (2013–2018), Zhang et al. (17) found that PNI was significantly negatively correlated with the risk of diabetic kidney disease and all-cause mortality in type 2 diabetes mellitus patients. A critical PNI threshold of 50.5 was identified, below which patients faced increased risks of both outcomes (17). Xu et al.’s (19) study in elderly populations with COPD found that lower PNI was associated with increased all-cause mortality. A study analyzed data from the NHANES between 2007 and 2018 to investigate the relationship between PNI and mortality risk in patients with gestational diabetes mellitus (GDM). The findings indicate that each unit increase in PNI is associated with a 9% reduction in all-cause mortality risk among GDM patients. Furthermore, the study identified a critical PNI threshold of 50.75, below which the mortality risk is higher, while above this threshold, the risk is significantly reduced (20). Notably, our study’s larger sample size and broader population base provide more robust evidence for PNI’s utility as a prognostic indicator. While previous studies focused on specific disease populations (CKD, diabetes, and COPD), our investigation of the general U.S. adult population reveals the broader applicability of PNI as a mortality risk predictor. The non-linear relationship we identified, with plateauing effects above certain thresholds, aligns with findings from previous studies, though our identified thresholds differ slightly. Our observation of powerful protective associations in liver disease patients adds a novel dimension to the existing literature, as this specific subgroup analysis was absent in previous studies. The consistency of our findings across various demographic and clinical subgroups, coupled with the larger scale and comprehensive adjustment for confounders, strengthens the evidence base for PNI’s role in mortality risk assessment.

The underlying mechanisms for the negative correlation between PNI and mortality can be attributed to several factors. Firstly, malnutrition often leads to a compromised immune response, increasing susceptibility to infections and other complications that can exacerbate cardiovascular conditions (21). Additionally, malnutrition is associated with systemic inflammation, contributing to CVD progression (22). The inflammatory response can lead to endothelial dysfunction, a precursor to atherosclerosis and other cardiovascular events, thereby increasing mortality risk (23). Moreover, the relationship between PNI and mortality is particularly pronounced in elderly populations and those with chronic conditions. For example, studies have shown that elderly patients with low PNI value scores face significantly higher mortality risks due to the interplay of malnutrition, frailty, and comorbidities (24). This demographic often experiences a higher prevalence of malnutrition, which can exacerbate existing health issues and lead to poorer clinical outcomes (25).

The more prominent PNI associations observed in patients with liver disease and hyperuricemia can be explained through several evidence-based mechanisms. In liver disease patients, hepatic dysfunction directly compromises albumin synthesis, which is a core component of PNI calculation. Research demonstrates that PNI correlates closely with liver function, with PNI having important prognostic value in patients with chronic liver disease (26). Furthermore, PNI combines serum albumin levels and lymphocyte counts, serving as a validated tool for assessing immune-nutritional status (27). In liver disease patients, both immune function and nutritional status are simultaneously compromised, making PNI a particularly important predictor of disease severity and mortality. Regarding hyperuricemia patients, hyperuricemia is strongly linked to CVD risk, including hypertension, coronary artery disease, arrhythmia, and heart failure (28). Studies demonstrate that even slight increases in serum uric acid levels are independent risk factors for all-cause and cardiovascular mortality (29). Under metabolic stress conditions such as liver disease and hyperuricemia, the integration of nutritional and immune parameters reflected by PNI may better capture overall physiological reserve and mortality risk. Research indicates that PNI reflects immune nutrition and inflammation status, with low PNI values consistently associated with poor outcomes (30). Therefore, in these specific disease states, the prognostic value of PNI is amplified, demonstrating stronger associations with mortality outcomes.

Our study has several notable strengths. First, we utilized data from NHANES, a well-established nationwide database with standardized collection protocols and rigorous quality control measures. Notably, using survey weights in our analyses allows our findings to be representative of the broader U.S. adult population, enhancing the generalizability of our results. Second, our study included a large sample size with diverse demographic characteristics, providing sufficient statistical power to detect meaningful associations and conduct robust subgroup analyses. Third, we implemented comprehensive adjustment strategies for multiple potential confounders, including socioeconomic factors, demographic characteristics, comorbidities, and laboratory parameters, strengthening our findings’ validity. Fourth, the extended follow-up period allowed for a thorough assessment of long-term outcomes and mortality risks. Finally, our findings remained consistent across various sensitivity analyses and subgroup examinations, supporting the robustness of the observed associations between PNI and mortality outcomes.

Nevertheless, several limitations should be acknowledged. First, although NHANES provides comprehensive nationally representative data with standardized collection protocols, the cross-sectional nature of the baseline measurements means that changes in PNI over time could not be assessed. Second, while we conducted extensive adjustments for multiple potential confounders and performed various sensitivity analyses, as with all observational studies, the possibility of residual confounding cannot be eliminated. Third, although our study included a large sample size with diverse demographic characteristics, the findings may not be fully generalizable to populations outside the United States or specific clinical settings. Finally, while the follow-up period provided robust mortality data, future studies with even longer follow-up durations might reveal additional insights into the long-term prognostic value of PNI.

Our findings have several important clinical implications. First, PNI represents a readily available and practical tool for risk stratification in clinical practice, as it can be easily calculated from routine laboratory parameters (serum albumin and lymphocyte count). Second, our results suggest that healthcare providers should pay particular attention to patients with low PNI values, as they may be at increased risk for adverse outcomes. Regular monitoring of PNI could help identify high-risk patients who might benefit from more intensive surveillance and early intervention. Third, given the strong association between PNI and mortality outcomes, nutritional intervention strategies should be considered for patients with low PNI value scores. These interventions might include dietary modifications, nutritional supplementation, and lifestyle changes. Finally, the observed differences in PNI associations across various subgroups suggest the need for personalized approaches to nutritional assessment and intervention strategies, taking into account individual patient characteristics and comorbidities.

## 5 Conclusion

In conclusion, this large-scale, population-based study demonstrates a significant association between PNI and mortality outcomes in the U.S. adult population. Our findings indicate that an increase of one unit in PNI is associated with a 5% reduction in all-cause mortality and a 6% reduction in cardiovascular mortality risk. The critical threshold values were identified at 50.24 and 51.50, respectively. The protective associations were particularly pronounced among individuals with liver disease, and a clear dose-response relationship was observed across PNI quartiles. These associations remained consistent after comprehensive adjustment for potential confounding variables across various demographic and clinical subgroups. Our findings indicate that PNI may serve as a valuable indicator for risk stratification in clinical practice. Further prospective studies are required to validate these findings and assess whether interventions to improve PNI can effectively modify mortality risk in high-risk populations.

## Data Availability

The raw data supporting the conclusions of this article will be made available by the authors, without undue reservation.

## Footnotes

1
https://wwwn.cdc.gov/nchs/nhanes/default.aspx

2
http://www.Rproject.org
