# Supplementary material for: Association of prognostic nutritional index with all-cause mortality and cardiovascular mortality: a nationwide population-based cohort study
Source: Front Nutr. 2025 Jul 14;12:1530452. doi: 10.3389/fnut.2025.1530452 (PMC12302918; doi:10.3389/fnut.2025.1530452)
Supplement: Supplementary file 1 [file Supplementary_file_1.docx]

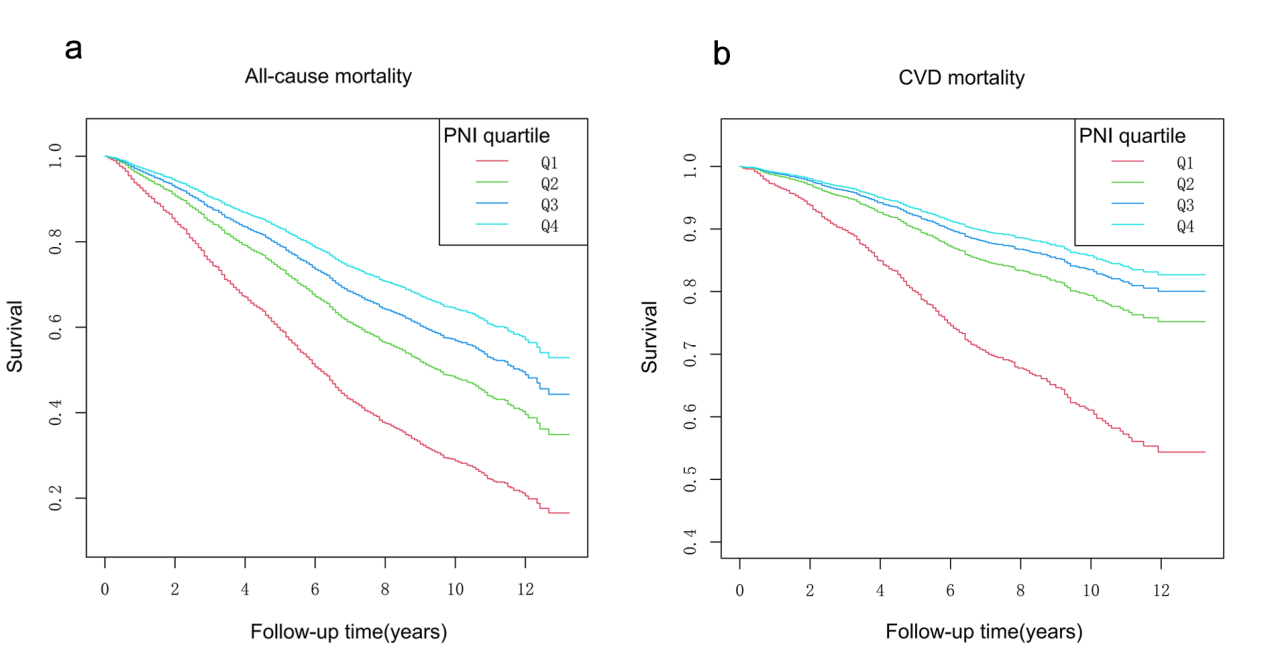


**Figure S1.** Kaplan-Meier survival curves stratified by PNI quartiles in CKD.Panel (a) shows all-cause mortality and panel (b) shows CVD mortality over 13 years of follow-up.


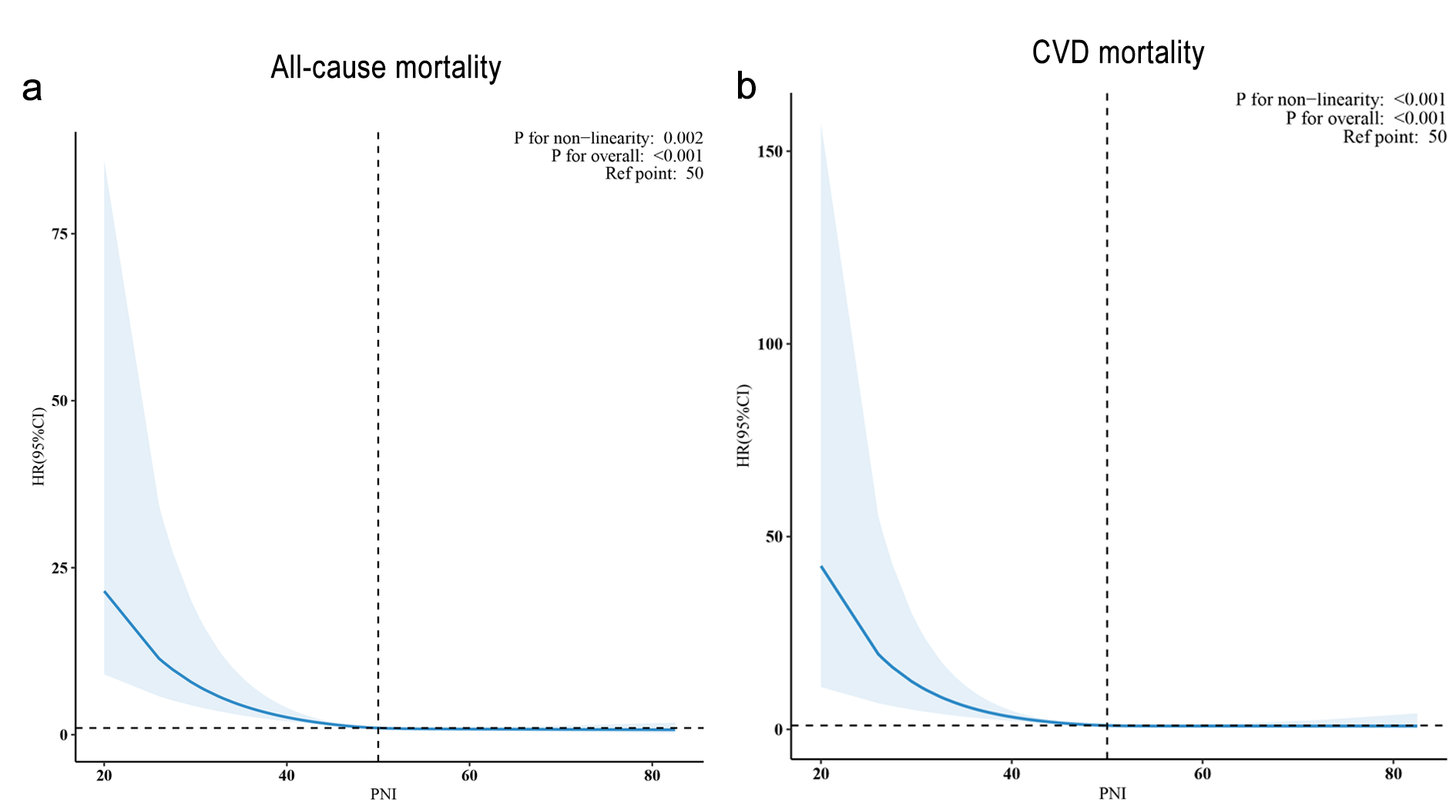


**Figure S2.**Non-linear association between Prognostic Nutritional Index (PNI) and hazard ratios (HRs) for all-cause and cardiovascular mortality in CKD. Restricted cubic spline curves showing the relationship between PNI and adjusted HRs with 95% confidence intervals (shaded areas).
